# Recommendations for Designing Health Information Technologies for Mental Health Drawn From Self-Determination Theory and Co-design With Culturally Diverse Populations: Template Analysis

**DOI:** 10.2196/23502

**Published:** 2021-02-10

**Authors:** Vanessa Wan Sze Cheng, Sarah E Piper, Antonia Ottavio, Tracey A Davenport, Ian B Hickie

**Affiliations:** 1 Brain and Mind Centre The University of Sydney Sydney Australia

**Keywords:** mental health, health information technologies, self-determination theory, eHealth, internet, digital health, adolescent, mental health services, young adult, LGBTQ persons, mobile phone, rural health

## Abstract

**Background:**

Culturally diverse populations (including Aboriginal and Torres Strait Islander people, people of diverse genders and sexualities, and culturally and linguistically diverse people) in nonurban areas face compounded barriers to accessing mental health care. Health information technologies (HITs) show promising potential to overcome these barriers.

**Objective:**

This study aims to identify how best to improve a mental health and well-being HIT for culturally diverse Australians in nonurban areas.

**Methods:**

We conducted 10 co-design workshops (N=105 participants) in primary youth mental health services across predominantly nonurban areas of Australia and conducted template analysis on the workshop outputs. Owing to local (including service) demographics, the workshop participants naturalistically reflected culturally diverse groups.

**Results:**

We identified 4 main themes: control, usability, affirmation, and health service delivery factors. The first 3 themes overlap with the 3 basic needs postulated by self-determination theory (autonomy, competence, and relatedness) and describe participant recommendations on how to *design* an HIT. The final theme includes barriers to adopting HITs for mental health care and how HITs can be used to support care coordination and delivery. Hence, it describes participant recommendations on how to *use* an HIT.

**Conclusions:**

Although culturally diverse groups have specific concerns, their expressed needs fall broadly within the relatively universal design principles identified in this study. The findings of this study provide further support for applying self-determination theory to the design of HITs and reflect the tension in designing technologies for complex problems that overlap multiple medical, regulatory, and social domains, such as mental health care. Finally, we synthesize the identified themes into general recommendations for designing HITs for mental health and provide concrete examples of design features recommended by participants.

## Introduction

### Barriers to Accessing Mental Health Care for Young Nonurban Australians

Around 1 in 5 Australians experience mental ill health regardless of whether they live in an urban or a nonurban (ie, regional, rural, or remote) area [1]. However, compared with urban Australians, nonurban Australians experience worse mental health outcomes, such as higher self-harm, suicidal ideation, and suicide attempt rates [2]. Nonurban Australians experience many barriers to accessing services and supports, such as cost, distance, a relative lack of service providers, stigma, and an increased emphasis on privacy [3,4].

These barriers can lead to limited access to mental health care for young people living in nonurban areas. Notably, rural young Australians discontinue prematurely from care at higher rates than those from urban or regional areas [5]. As service provision declines with distance from major cities [6], the lack of public transportation in rural and remote communities increases young people’s reliance on their supportive others (eg, parents) to take them to a service [7]. This reliance on others to access a service also raises the issue of anonymity, which can be problematic given the higher levels of stigma related to mental health issues in nonurban communities [4]. Furthermore, service opening times (often limited to weekdays during 9 AM to 5 PM), extensive wait times, and cost can add to these barriers to access mental health care [8].

### Culturally Diverse Young People

For culturally diverse young nonurban Australians, such as Aboriginal and Torres Strait Islander people, people of diverse genders and sexualities, inclusive of and not limited to lesbian, gay, bisexual, transgender, queer, intersex, asexual, questioning, and pansexual people (henceforth referred to as LGBTQIA+ people in this paper), and culturally and linguistically diverse (CALD) people, multiple forms of inequality often intersect to create compounded barriers in the form of decreased mental health literacy, financial barriers, increased social and self-stigma, and a lack of mental health services compounded with geographic inaccessibility [7]. These culturally diverse groups experience both poorer mental health outcomes and reduced access to mental health care [9-13].

Barriers to accessing mental health care for Aboriginal and Torres Strait Islander people living in nonurban areas include a lack of trust in health services, lack of culturally appropriate care, and lack of available services in remote areas [10,14-16]. For young LGBTQIA+ people, reported barriers include concerns that health professionals would not be able to cater to an individual’s specific identity or needs [17], a fear of experiencing homophobia and/or transphobia, and a reluctance to come out to a health professional [18]. Finally, it has been reported that young CALD people, including young people from migrant and refugee backgrounds, underutilize the public mental health system in Australia [19,20]. The key barriers to mental health care access for young CALD people include the stigma associated with mental illness, concerns regarding confidentiality, limited knowledge of available services, language barriers and communication difficulties, fear of discrimination, and a lack of trust in service providers [19,21]. These barriers to appropriate care for these 3 populations can be exacerbated in a rural setting because of fewer available culturally competent services and the increased emphasis on privacy within close-knit rural communities [4,7].

### Technologies to Empower Mental Health Care Access

The term health information technology (HIT) has been defined as the “application of information processing involving both computer hardware and software that deals with the storage, retrieval, sharing, and use of health care information, data, and knowledge for communication and decision making” [22]. HITs have immense potential to address intersecting barriers to mental health help seeking in nonurban areas. They can provide individuals with access to mental health services regardless of geographical location, vulnerability, or socioeconomic status [23]; reduce the wait time to access mental health support; and remove constraints around service opening hours [24]. HITs can also make psychoeducation and help-seeking sources easier to locate and access [23]. Not only can HITs enable access to care, but they can also enhance the care delivered by mental health services, for example by using synchronous communication protocols such as video chat and real-time data tracking through apps and other software. However, if HITs do not achieve a minimum standard of usability (or ease of use) as well as user experience, they experience suboptimal uptake and dropout rates [23,25]. Given the varying levels of satisfaction (or lack thereof) of young people toward web-based mental health resources [26], it is important to ensure that HITs support the needs of their users appropriately.

To investigate how HITs can improve and transform mental health care in Australia, the University of Sydney’s Brain and Mind Centre (BMC) established the Youth Mental Health and Technology Program (YMH and Tech Program). As part of this program, an HIT prototype known as the BMC Youth Platform was developed to enhance the quality of health care provided by traditional mental health services. The BMC Youth Platform consists of a set of web-based personalized clinical assessments and longitudinal tracking tools for young people to monitor psychological, neurocognitive, social, and medical characteristics and plan individualized and more effective longer-term interventions. It aims to support the delivery of mental health services and the management of mental health symptoms and as such is to be used by young people, their health professionals, and their supportive others. A summary of the key BMC Youth Platform components is presented in Table 1.

**Table 1:** Key components of the Brain and Mind Youth Platform.

Purpose	Component
Start page	Start page
Record of basic demographic information	About me page
Record of medical history	Health history page
Clinical assessment	Questionnaires
Holistic multidimensional tracking of mental health	Dashboard of multiple health cards (each representing a different health domain)
Recommended interventions	Care options

The BMC Youth Platform was implemented into 5 *headspace* centers (a primary mental health service for young people aged between 12 and 25 years) in urban locations within Sydney, New South Wales, and learnings from these implementations included the importance of ongoing co-design with end users to ensure iterative improvements to the HIT [24]. Our past research has also confirmed the suitability of, and the potential to further explore, the use of co-design methodologies when implementing HITs in nonurban areas of Australia. Crucially, co-design methodologies have been found to facilitate the implementation of more acceptable digital solutions for mental health in nonurban areas that take local circumstances (such as natural disaster rates) into account [27].

### Iteratively Co-designing and Testing HITs

The BMC Youth Platform is the product of multiple years of co-design and user testing conducted, as part of the YMH and Tech Program, with various representative end user groups, including young people aged between 16 and 25 years and health professionals [28,29]. Co-design (also referred to as participatory design) is a key research methodology that enables the perspectives and preferences of the target end user population to influence subsequent development of the HIT [30]. When conducted appropriately, participatory design is effective in obtaining insights from population groups that are marginalized or otherwise affected by structural inequalities [31,32] and results in higher levels of end user acceptability of the final intervention [33].

One product of the YMH and Tech Program is the Project Synergy Research and Development Cycle, which applies co-design methodologies to the design, development, implementation, and feasibility testing of apps and technologies [28]. In total, 3 key principles underpin this cycle: involving target end user populations (including, but not limited to, young people, supportive others, health professionals, and other service staff) as active participants throughout the entire design process, treating young people as design partners, and continually and iteratively evaluating the acceptability of the technology from the perspective of its target audience.

Although previous co-design and user testing work done as part of the YMH and Tech Program focused on the needs of young Australians in urban areas [28] and expanded them to young Australians in nonurban areas [27], these methodologies have not yet been applied to culturally diverse young Australians in nonurban areas.

### Objectives

This study aims to identify how best to improve an HIT (such as the BMC Youth Platform) for culturally diverse young Australians in nonurban areas and to synthesize findings into recommendations for designing HITs for mental health.

## Methods

### Ethical Approval

The University of Sydney’s Human Research Ethics Committee (protocol number 2018/130) approved this research study before the start of data collection.

### Inclusion Criteria

To satisfy the inclusion criteria for participation in the study, participants were required to:

- be aged 12 years or above
- be either a young person attending a participating *headspace* center; a supportive other of a young person attending a participating *headspace* center (eg, family member, caregiver, friend); or a health professional, service manager, or administrator working at a participating *headspace* center
- be proficient in reading and speaking English
- complete the participant consent process.

### Participants

*headspace* staff advertised this study using posters and postcards distributed throughout the participating centers. Recruitment was passive to avoid any perceived coercion, whereby a potential participant would contact the research officer listed on the advertisements to express their interest in the study and request further information. Upon the potential participant’s request, the research officer would then forward (via email) on the participant information statement, participant consent form, and screening survey to determine eligibility.

For participants under the age of 15 years, both the young person and their guardian were given detailed and age-appropriate information about the research study before the workshop via a parental information statement and a child assent form. At the beginning of the workshop, research officers reminded all participants about what the workshop would involve, provided an opportunity to ask any questions, and reminded them that the participation was voluntary. The research officers spoke separately to participants under the age of 15 years and their guardians to ensure that they understood what the workshops would involve and what the study was about. They also answered any questions and reminded the young person that they could withdraw from the study at any time without consequence. If a young person agreed to participate in the workshop, their guardian provided a signed parental consent form and the young person provided a signed child assent form.

The young people and their supportive others were reimbursed with a gift card valued at Aus $30 (US $23) for study participation.

### Co-design Workshops

#### Workshop Location and Demographics

A total of 10 co-design workshops were held from July to September 2018 in *headspace* centers across the Australian states of New South Wales, South Australia, and Queensland.

We initially invited *headspace* centers to participate in this study, trying to ensure a representation of centers located in nonurban areas. Participating *headspace* centers were aware of the possibility of implementing this technology in their centers in the future. Although researchers did not specifically recruit Aboriginal and Torres Strait Islander people, LGBTQIA+ people, and CALD people, because of local population demographics, these groups were naturalistically represented in the participant sample.

#### Workshop Protocol

**Figure 1:**
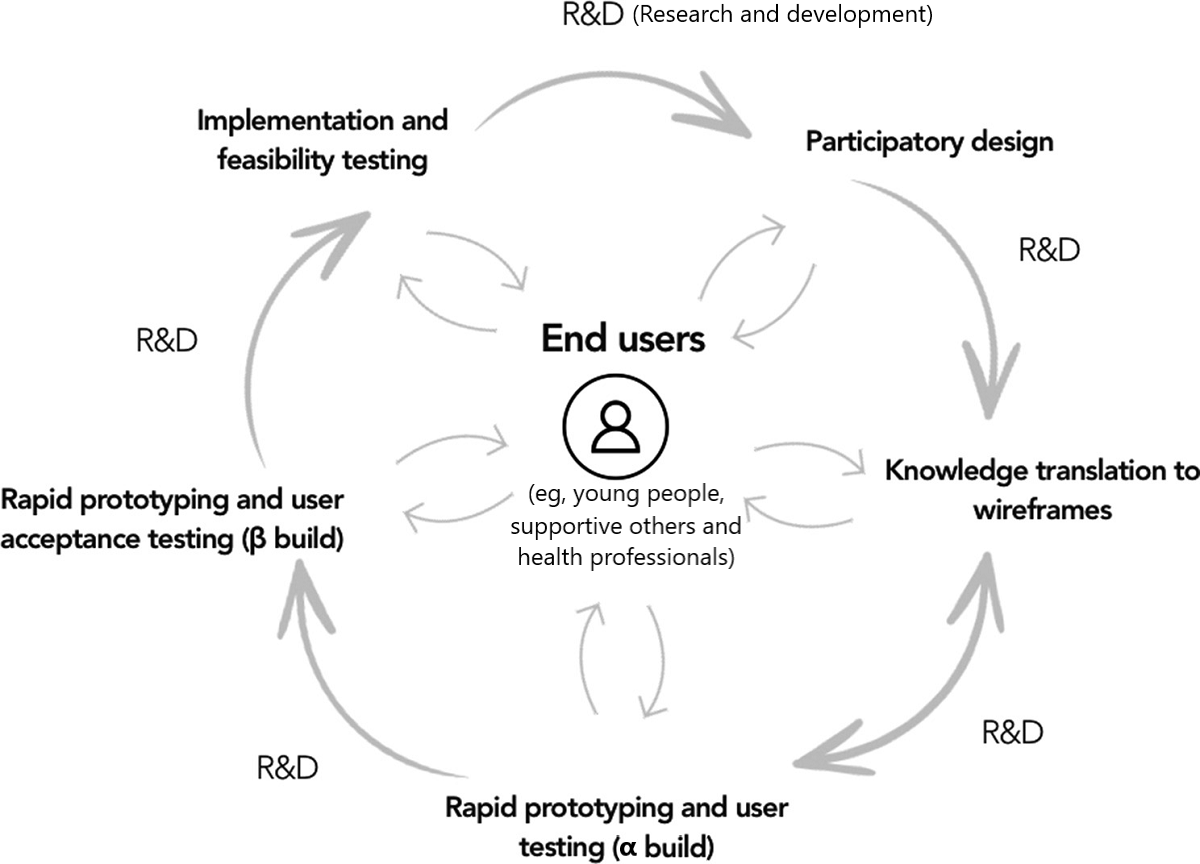
Project synergy research and development cycle.

These workshops represent phase 1 of the Project Synergy Research and Development Cycle (Figure 1 [28]), where co-design workshops are rapidly conducted across different sites until theme saturation [28]. In these workshops, technology designs, ideas, and principles are evaluated by participants, sometimes iteratively (if enough time has passed for insights from a previous workshop to be translated into a testable wireframe or prototype). Previous work describing the process and outcomes of this methodology [28,29,34,35] as well as work on a separate mental health and well-being app [36] have been published.

Although general workshop agendas were adhered to as appropriate, facilitators (the second, third, and fourth authors) also conducted workshops flexibly and followed up on topics in response to individual workshop dynamics. Certain topics were also given more priority to explore in different locations and contexts (eg, technology use and connectivity in nonurban areas). Therefore, the workshop content and outcomes varied slightly among workshops.

After each workshop, facilitators reviewed the workshop findings, adapting the general workshop agenda to remove data-saturated topics and add new and further topics of interest. An example of a workshop agenda is provided in Multimedia Appendix 1.

Owing to the number of participants and other contextual factors, workshops ranged from 2.5 to 4 hours in duration and consisted of the following stages: discovery, evaluation, and prototype. In the discovery stage, facilitators led discussion around the following topics: general technology use, technology use for the purposes of supporting health and mental health, and internet use and connectivity. In the evaluation stage, participants were presented with paper printouts of various components of the BMC Youth Platform and asked to annotate them with their thoughts and comments. Finally, in the prototype stage, participants were asked to brainstorm new ideas, functionalities, and wireframes (with marker pens and sketchbooks) for the BMC Youth Platform. Owing to the sensitivity of the subject matter, workshops were not audiorecorded or videorecorded to decrease the risk of identification and facilitate participant disclosure. Instead, scribes took notes at each workshop. All workshops included at least one facilitator who was appropriately qualified in mental health to provide counseling support to the participants if needed.

### Knowledge Translation

All workshop data, including notes and artifacts from the evaluation and prototype stages, were collated and reviewed by an independent knowledge translation team consisting of 2 young people (listed in the Acknowledgments) who had never previously been exposed to the BMC Youth Platform or its concepts. The knowledge translation team summarized the outcomes of the discovery and prototype stages and conducted a procedure similar to descriptive content analysis [37] on the outcomes of the evaluation stage. Specifically, all annotations were reviewed by each team member, who noted their general observations. They then coded the annotations together, organizing codes according to semantic themes representing different components of the BMC Youth Platform (Table 1).

### Template Analysis

To supplement knowledge translation insights and identify general recommendations on designing HITs for mental health, we conducted a type of codebook thematic analysis [38] known as *template analysis* [39,40] on the workshop data. Specifically, the data set consisted of the knowledge-translated, summarized outcomes of the evaluation stage (in tally form) and scribe notes from each workshop. In recognition of the fact that generic thematic analysis (including template analysis) is a method with many different approaches that reflect a wide variety of epistemological (theory of knowledge) and ontological (theory of being) assumptions [38], we located our approach within a philosophical position of qualitative neopositivism. This position assumes a realist ontology and epistemology.

The data set was coded with NVivo 12 (QSR International). The first author initially coded all the data using a bottom-up, descriptive approach at a level close to the data (eg, *avoid clinical jargon* and *worry that information is confronting*). At the conclusion of this first round, the first author determined that certain groups of codes fit well with self-determination theory [41,42]. Although self-determination theory is originally a theory of intrinsic motivation, human-computer interaction research has found it to be applicable to user engagement with digital technologies [43,44]. Self-determination theory has also been successfully applied to user needs, facilitators, and barriers for mental health technologies [45,46].

Using self-determination theory as a partial reference for a priori themes, the first author grouped these descriptive codes into a preliminary coding template. A second coder (the second author) then independently coded 10% of the data according to this preliminary coding template to check its quality. In this case, quality was defined as the clarity of definitions and whether the template comprehensively covered the data set [40]. Both coders then discussed all discrepancies in coding until they were resolved, and insights from this process were used to refine the coding template. This iterative process of independent coding, comparison, and refinement of the coding template was repeated until a satisfactory level of interrater reliability was reached (Cohen kappa for all codes >0.65), which took 3 rounds of coding. Following this, the final interpretation of the template in relation to addressing our study aim was conducted.

## Results

### Participants

Table 2 reports workshop details, including dates, location, and participant characteristics. All workshops except workshop 5 (Ashfield, New South Wales) were conducted in nonurban areas. All *headspace* clients are young people aged between 16 and 25 years unless specified otherwise. Staff included mental health clinicians (psychologists, mental health nurses, and social workers), youth workers, administrative staff, and center managers from *headspace*.

**Table 2:** Workshop and participant details (N=105).

Workshop number	Workshop date	Location	Facilitators, n	Participants, n	Participant demographic information		
					Gender	Participant role	Cultural or personal identification^a^
1	June 18, 2018	Edinburgh North, South Australia	3	10	8 female and 2 male	6 clients, 1 support person, and 1 staff member	None disclosed
2	June 20, 2018	Edinburgh North, South Australia	3	11	7 female and 4 male	5 clients, 3 support people, and 3 staff members	1 CALD^b^
3	July 20, 2018	Broken Hill, New South Wales	3	9	4 female, 4 male, and 1 transgender	6 clients, 1 younger client aged between 12 and 15 years, and 2 staff members	2 CALD and 2 Aboriginal or Torres Strait Islander people
4	July 25, 2018	Townsville, Queensland	2	11	6 female and 5 male	8 clients and 3 staff members	2 Aboriginal or Torres Strait Islander people and 5 LGBTQIA+^c^ people
5	August 9, 2018	Ashfield, New South Wales	3	5	4 female and 1 male	3 clients, 1 younger client aged between 12 and 15 years, and 1 support person	5 CALD
6	August 14, 2018	Wagga Wagga, New South Wales	2	9	7 female and 2 male	2 clients, 2 younger clients aged between 12 and 15 years, 3 staff members, and 2 support people	None disclosed
7	August 22, 2018	Bathurst, New South Wales	2	11	5 female and 6 male	6 clients, 4 younger clients aged between 12 and 15 years, and 1 staff member	None disclosed
8	August 28, 2018	Orange, New South Wales	2	22	6 female and 16 male	7 clients, 11 younger clients aged between 12 and 15 years, and 4 staff members	5 Aboriginal or Torres Strait Islander people
9	August 30, 2018	Wollongong, New South Wales	2	7	4 female, 2 male, and 1 gender neutral	6 clients and 1 staff member	1 person with disability
10	September 4, 2018	Dubbo, New South Wales	2	10	7 female, 2 male, and 1 gender neutral	7 clients and 3 staff members	4 LGBTQIA+

^a^Participant demographic information, particularly gender and cultural or personal identification, was provided by participants on a voluntary basis and was therefore unable to be captured consistently across workshops.

^b^CALD: culturally and linguistically diverse people.

^c^People of diverse genders and sexualities, inclusive of and not limited to lesbian, gay, bisexual, transgender, queer, intersex, asexual, questioning, and pansexual people.

### Template Analysis

**Figure 2:**
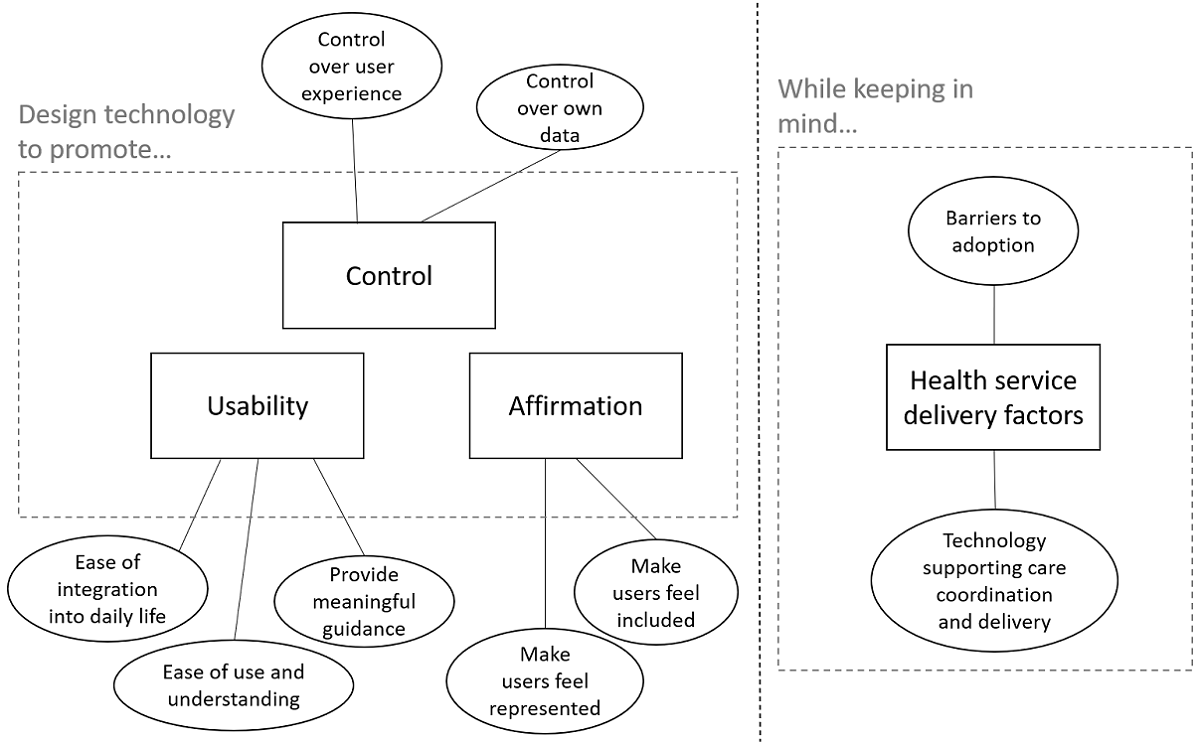
Final coding template.

Figure 2 shows the coding template following analysis and the refinement of the codes. During the analytical process, numerous codes were generated from the data set. These codes were organized into 11 low-level themes, which were then organized into the following high-level themes (design principles): control, usability, affirmation, and health service delivery factors. The full coding template (including a codebook of definitions) is presented in Multimedia Appendix 2.

Although many participants belonged to culturally diverse groups, they made many design recommendations applicable to universal human experience. These recommendations overlapped closely with the 3 basic psychological needs postulated by self-determination theory, namely, autonomy (corresponding to our theme of control), competence (corresponding to our theme of usability), and relatedness (corresponding to our theme of affirmation) [41,42].

### Give Users Control

First, participants emphasized the importance of having control over their user experience and their data.

#### Control Over User Experience

Participants made clear their expectations that any technology they use should give them the ability to control their own user experience, for example, by active customization of key design elements such as background color, layout, and the ability to upload an avatar. Participants from CALD backgrounds and participants with disabilities also expressed a desire to be able to change language and font size and to be able to consume information in different audiovisual formats (eg, text-to-speech options).

This desire for control also extended to the care being delivered to young people, with participants appreciating that they could make informed choices on which care options to embark on to progress their care. Participants also wanted to be able to arrange the health cards on their dashboard in ways that would make more sense or that they would find less overwhelming:

How the cards are ordered is important, [it] would be great to have the cards you want to work on together or first, group cards by color.

Bathurst, young person

Can you collapse aspects if [you’re] feeling “shitty”?

Wagga Wagga, young person

Finally, participants also suggested providing mechanisms through which user feedback could be provided and actioned on, for example, through a dedicated feedback section.

#### Control Over Data

Participants also stressed the importance of having control over their own data at all times, not only through data security (authentication and encryption, eg, through password or personal identification numbers) but also through data privacy and sharing. A key feature of the BMC Youth Platform is that young persons’ dashboards of results are visible to health professionals at their service. The young persons can also share their dashboards with trusted supportive others, such as parents or friends. Although participants found it acceptable to share their data in this form with their health professionals, they were more hesitant to share their data with their supportive others (eg, parents or friends). Participants wanted fine-grained control and the ability to grant or revoke their supportive others’ access to certain health cards, particularly those deemed by young people to be more sensitive, such as substance and tobacco use:

I don’t want mum to see smoking, but they can see anxiety.

Edinburgh North, young person

The consent and data sharing status of each of their health cards as well as other parts of their data (such as health history) would also need to be presented to the user clearly and transparently:

How long do they have access to the information? [It] needs expiry, for example when [you] repeat the questions, or between 1-30 days, can check in to add or remove people.

Wollongong, young person

Privacy setting on cards can be set with clinician at first appointment, then the parent or carer receives the email invitation. They might have their own dashboard to look at.

Dubbo, headspace staff

### Make the Technology Usable

Second, participants expressed that the technology should not be difficult to use, that is, it should (1) be easy to use and understand, (2) provide meaningful guidance, and (3) be interoperable.

#### Ease of Use and Understanding

Participants specified that the technology should be easy to use and understand at first glance and that it should not be overwhelming or frustrating to use. The technology should be clear, unambiguous, and consistent in language and user interface (including layout, icons, and imagery), and clinical jargon should be avoided. The latter point was especially relevant for younger audiences aged between 12 and 15 years because of lower reading levels. However, although participants found clinical terms such as “social and occupational functioning” and “psychosis-like experiences” confusing and intimidating, it was difficult to come up with alternative, more acceptable terms. After considering the issue, many participants suggested using direct language and providing definitions.

#### Provide Meaningful Guidance

Participants further specified that the technology should present information in meaningful chunks or sequences (eg, displaying participants’ data on the most important health domains first) to promote a gradual understanding of psychoeducational concepts. This was particularly the case for the onboarding stage, which was where many participants would encounter clinical terminology and their own data for the first time. In addition to presenting information in a staggered, chunked manner, the technology should also provide meaningful guidance on how to use and interpret the technology. Participants overwhelmingly endorsed a model of delivering information whereby a page was kept simple and clutter-free at first presentation (relating to the previous tenet of ease of use and understanding), with users being given the freedom to learn more via a *more information* button or by hovering over complex terms (eg, *mania*, *psychosis*, or *clinician*).

Similarly, participants expressed that the technology should provide prompts or mechanisms through which users could gain an understanding of how to use the technology and how the technology could help them, for example, via a help button or Frequently Asked Questions (FAQ) page or through prompts reminding them to check their dashboard and complete new psychometric questionnaires at appropriate time intervals. These prompts could be tailored based on their questionnaire responses:

If a health card is red, the health professional (or system) should keep close eye on this—and prompt you to repeat the questionnaire.

Dubbo, headspace staff

#### Interoperability

Participants also emphasized that any HIT should be easy to integrate into daily life. This technology should be able to compile data from other apps (eg, step tracking apps) and enable all information of a user to be accessed from one location. Similarly, some participants endorsed the idea of quick, convenient authentication via social media, although others were concerned about data security issues. Young people overwhelmingly expressed a preference for mobile usability (responsive web design) and mobile integration (eg, via SMS text messaging instead of email), as many of them had neither easy access to computers nor an email address.

### Affirm Users’ Identity and Preferences

Finally, participants emphasized the importance of an HIT making its users feel represented and valued.

#### Make Users Feel Represented

Despite one of the purposes of HITs being the facilitation of users’ mental health care via routine outcome monitoring, participants (both health professionals and young people) felt that it should provide functionality to represent the user beyond traditional clinical information such as demographics and mental health status. Participants wanted to be able to define themselves in more nuanced ways and felt that the screenshots they were presented with reflected a technology that could not yet allow them to do so. Their suggestions for ways to define themselves comprehensively and accurately ranged from simple actions such as being able to specify their preferred pronoun and being offered a more diverse range of response options when specifying their demographics (eg, sexuality, gender, and ethnicity) to more comprehensive methods such as adding new health cards to provide more information on additional domains such as family violence and homelessness or elaborating on responses via free text. This was judged to be particularly relevant given that *headspace* also offers support for domains outside mental health (such as with work and study):

Dashboard needs to reflect what is important to me, [for example] culture, sexual health and homelessness.

Dubbo, young person

These questions are very mental health–focused but headspace does a lot more.

Bathurst, headspace staff

Some LGBTQIA+ participants also felt that their sexual identity and preferred pronouns should not “be hidden away” in a separate *About me* tab, as this made it harder for them to come out to their health professional, and that instead it should be reflected in the dashboard.

Participants also felt that HITs should cater to them. Not only should they be usable regardless of physical or mental ability (mental health status, disability, or other accessibility considerations) but they should also offer a personalized user experience and present their various components (questionnaires, care options, psychoeducation, language, etc) in ways that are tailored to their users’ personal circumstances (eg, gender, culture, location, and questionnaire responses):

[It] should be designed like Spotify, when you select your music it also suggests other artist in the same sort of music. [On the BMC Youth Platform] if [you] choose tobacco it would also give you the other drug [questions] like alcohol and cannabis.

Bathurst, young person

Tabs at top [could link to psychoeducation resources]—possibly an option of “how to talk to my parents,” “how to talk to my partner,” [that is] some ideas about what you can do with this information in the meantime. It should be tailored to who the login “user” is, [that is] at a certain age it may be directed to something different than to a 13-year-old, or a parent.

Edinburgh North

Finally, the CALD participants emphasized that the technology should cater to their needs, namely, that their languages should be supported and that the technology should acknowledge that different cultures could approach mental health differently, for example, by viewing them as “life circumstances” instead of “mental illness.”

#### Make Users Feel Valued

Both health professionals and young people were adamant that an HIT user should not feel reduced to a diagnosis or an assortment of diagnostic labels. Instead, the HIT should be designed to celebrate nonclinical aspects of personhood as well, such as a user’s likes and aspirations (eg, via a *My Goals* section). Although these aspirations could relate to a user’s mental health (eg, improving their mood or sleep), they could also reflect a user’s other goals. Participants also suggested having a dedicated space for users to store personally meaningful resources, such as a journal, pictures of inspirational people, or self-care resources useful to them:

If clinicians can see this page [they] will have a more positive approach to the relationship [and] will be able to connect with the young person.

Bathurst, young person

Participants also raised concerns that the screenshots of the technology they were presented with could potentially frame users too negatively. Instead, they preferred a strengths-based, positive framing approach that provided positive affirmations, kept “users on track,” and celebrated achievements and strengths. Participants also recommended adding gamification to the technology to increase motivation to engage and promote a sense of progress, for example, via markers of achievements (eg, ribbons or “becoming a BMC Youth Platform warrior”) or a leveling and reward system. They further specified that any gamification should “reward effort more than outcome,” that is, rewards should be assigned based on the level of long-term engagement and the extent to which the user has explored (or completed) the technology.

An HIT should also not alienate the user and make them feel alone in their struggles with mental health. Instead, the technology should promote a sense of social connection (even parasocial connection), for example, by providing testimonials and links to other people’s mental health stories (eg, an “others that struggle with this issue” section) and by normalizing suboptimal mental health status (eg, “It’s OK to feel bad”).

### Health Service Delivery Factors

In addition to the more universal design principles described above, participants also contributed insights into what could broadly be categorized as health service delivery factors. This included perceived barriers to the widespread adoption of HITs for mental health care and how HITs could be used to support mental health care coordination and delivery.

#### Barriers to Adopting HITs for Mental Health Care

Although this was not the key topic of investigation, during workshops, participants discussed barriers to adopting digital technologies for mental health care that were relevant to their regions and communities.

The limitations of internet connectivity in nonurban areas, raised in 7 of the 8 nonurban areas investigated, was the most commonly cited barrier, with slow internet speeds and a lack of access to home internet in certain geographic areas being the main issues. Participants were familiar with free Wi-Fi locations, such as libraries, local schools or universities, and fast-food restaurants. Using internet in nonurban areas was also associated with several costs that were unsustainable for young people, including the cost of purchasing smartphone apps as well as the cost of mobile data itself:

I use public Wi-Fi ([McDonald’s] or university Wi-Fi) to get internet since my home connection is bad.

Wagga Wagga, young person

Health professionals who participated in the workshops also raised the issue of software fatigue. Currently, *headspace* protocols mandate multiple other pieces of software to be used to manage minimum data entry for its clients. Health professionals were hence cautious of new technologies, stating that “[it’s a] real turn off to do the same questions more than once.” Instead, they would prefer that client responses could be “shared between systems” in the case of overlap:

With 96 clients on my list [I] do not want 96 notifications via email as there is enough to review with the current system.

Dubbo, headspace staff

#### Technology Supporting Care Coordination and Delivery

Collectively, workshop participants outlined concrete suggestions through which HITs could support health professionals and clients during mental health care.

First, HITs could help health professionals and clients understand the progress of the client’s care at all stages of the care. Before the first appointment, they could provide icebreakers to help potential clients become comfortable with their health professionals and provide health professionals with their client’s dashboard of results to allow them to prepare for the first appointment more effectively. This was viewed as critically important given that at the time of the workshops, the Australian public mental health system only funded 6 1-hour sessions with a psychologist (extendible to 10 sessions given further referral) per year:

It would take the whole session to go over history alone, with only 10 sessions [we] can’t afford to have a session devoted to not achieving anything.

Wollongong, young person #1

We need something [that isn’t] telling your story again to a new psychologist each time. It’s not schema therapy with 20 sessions, 6 sessions are precious.

Wollongong, young person #2

During the course of care, HITs could maintain a record of the client’s health over time and over a variety of domains (multidimensional routine outcome monitoring) as well as a record of care options attempted over time. This record could then be used as a reference to prompt topics of discussion between the health professional and client:

I want to see my apps linked into the system to share with headspace, so they can see my progress in real time.

Dubbo, young person

[On the system] buttons could say “what can I do now?,” “what to chat to my clinician about?”

Wagga Wagga

This record could be exported and transferred over to a new mental health service or a new health professional, should the client require it, to minimize administrative and psychological burden on the young person interacting with their new health professional:

Retelling your story is one of the hardest [things].

Wollongong, young person

By enabling tracking of a client’s progress during care, HITs could also support the health professional and client in gaining a more unified understanding of how to direct the client’s care. The health professional could tailor their support according to their clients’ needs in mental health and other domains. Similarly, by seeing how their self-reported health changed over time and following different care options, the client could make more informed choices on which health domains and care options to focus on.

Such technologies could also improve the efficiency and safety of clinical care. For example, video calling functionalities could overcome long distances in large rural health catchments and allow for more flexible appointment scheduling. Young people also expressed that they would find it easier to complete questionnaires on a device rather than answer them face-to-face with a health professional. Finally, such technologies could alert mental health services when their clients are experiencing emergency scenarios in real time, assisting them in risk management.

## Discussion

### Using Self-Determination Theory to Design HITs for Mental Health

The findings of this study can be broadly categorized into (1) design recommendations for HITs for mental health and well-being and (2) health service delivery factors to consider when designing such technologies. In other words, the former category reflects how such technologies should be *designed* and the latter category reflects how such technologies should be *used*.

The design recommendations approximately map to the 3 basic psychological needs proposed by self-determination theory: autonomy, competence, and relatedness [41]. As mentioned before, self-determination theory is a theory of motivation (including both intrinsic and extrinsic motivation) that has been applied to the design of health technologies [44,47] as well as web-based help seeking for mental health problems [45,46]. The fact that we found our workshop findings to align with self-determination theory constructs is a further support for its application to the design and delivery of HITs. Currently, many HITs do not explicitly apply theories of engagement or health behavior change to their design and delivery [48]. However, our results suggest that the contribution of these theories to HITs is equally important as that of evidence-based clinical content.

Importantly, although our core design recommendations map neatly onto the 3 basic psychological needs, how they are executed in practice can simultaneously support multiple needs [46]. For example, from our data, we found technological accessibility to be a blend of *usability* (ease of use), *control* (eg, customizing visual elements such as font size), and *affirmation* (inclusiveness) and avoiding clinical jargon to be a blend of *usability* (not using difficult terminology) and *affirmation* (avoiding making users feel reduced to a diagnosis). A comprehensive list of recommended design features identified from our workshops is shown in Table 3.

Our results are broadly consistent with previous research, reflecting the importance of promoting social connection [32,46], personalization and customization [36,46], clear and casual language [36], and addressing data security and privacy concerns [7,49].

Our results also suggest that any recommendations from participants should be evaluated through the lens of self-determination theory, with explicit consideration given to how each suggested change could promote each of the basic psychological needs. This is in line with previous research on applying self-determination theory to help seeking for mental health problems [45,46]. This measured evaluation is important given that many participant suggestions involved broad concepts or specifications and may reveal that further expertise needs to be consulted. For example, participants recommended implementing gamification to “reward effort more than outcome” and suggested several mechanics through which HITs could be gamified, including level-based reward systems and achievements. However, gamification is not merely a collection of gamification elements but instead the deliberate integration of gameful mechanics into a technology to support its core functionalities [50]. Implementing this suggestion, therefore, would involve not only the iterative user testing of the gamified HIT with the target end user population but also the consultation of gamification designers to integrate gamification at a deeper, systemic level that promotes both the HIT’s aims and user motivation to engage with the HIT (via the 3 basic psychological needs).

**Table 3:** Participants’ recommended design features and corresponding basic psychological needs.

Broad design category	Recommended design feature	Supported basic psychological needs
User interface and experience	Customizable user interface (language, colors, layout, font size, etc) to accommodate user preferences and needs	Autonomy and competence
User interface and experience	Tailored user experience	Relatedness
User interface and experience	Meets international accessibility standards (eg, web content accessibility guidelines)	Competence and relatedness
User interface and experience	Customizable information input and output (eg, being able to submit free text that more accurately describes you and being able to organize how your data are displayed) for ease of tracking and understanding	Autonomy, competence, and relatedness
User interface and experience	Clear layout, icons, and imagery	Competence
Content and functionality	Ability to provide user feedback that will be actioned on (eg, feedback or evaluation section)	Autonomy and relatedness
Content and functionality	Instructional prompts giving guidance on what to expect and how the technology will help the user and reminder prompts to promote reengagement	Competence
Content and functionality	Provision of optional additional information not core to the experience; however, the user can consult to learn more (eg, psychoeducation or a frequently asked questions section)	Autonomy and competence
Content and functionality	Gradual onboarding and the provision of information in meaningful chunks to support learning and understanding (eg, showing the most important health domains first on a health dashboard)	Competence
Content and functionality	Consider element of fun (eg, gamification)	Relatedness
Content and functionality	Promote social connection with peers and communicate to users that they are not alone (eg, through peer support groups and testimonials)	Relatedness
Language and tone	Adopt a strengths-based approach and celebrate nonclinical aspects of personhood such as likes, aspirations, strengths, and achievements	Relatedness
Language and tone	Cultural competence	Relatedness
Language and tone	Clear, casual, unambiguous, and consistent language that avoids clinical jargon and loaded terms	Competence and relatedness
Interoperability	Ease of integration with other apps and technologies (eg, health apps or convenient authentication methods)	Competence
Interoperability	Mobile integration as young people do not use email or computers frequently	Autonomy and competence
Security and privacy	Industrial-grade data security	Autonomy
Security and privacy	Fine-grained (individual level) data sharing functionality	Autonomy

Often, participant recommendations seemed to contradict each other at the surface level. For example, we observed tensions within participant recommendations on language, with an emphasis on increasing understanding (eg, by using clinically accepted terms and providing definitions when needed) conflicting with an emphasis on using casual, simple, and nonloaded language. Similarly, the focus on ease of use (by presenting information clearly and in a manner that is not overwhelming) superficially conflicted with the desire to have access to contextualizing information as well as the desire to improve representation by increasing the number of psychosocial domains represented on the dashboard of results. When this contradiction was raised, participants proposed the solution of giving users the ability to customize their own user experience and make it as simple, or as complex, as they liked. Under self-determination theory, this solution would allow each user to satisfy their needs for autonomy, competence, and relatedness at an individual level.

However, although many contradictions were solved in a way that was acceptable to our participants on an individual level, some solutions raise implications on the service level. For example, an overarching recommendation from the workshops that participants overwhelmingly agreed on was the desired ability to provide more nuanced information about oneself via free text. On the service level, however, this solution would raise implications for, and potentially conflict against, clinical safety protocols unless the free-text fields were moderated and constantly monitored (on a 24-hour basis) for any indication of suicide risk. Currently, the role of this moderator would most practically be filled by a health professional employed at the service, a human resource allocation luxury many publicly funded mental health services do not have. Although it is important to adhere to clinical safety protocols, this situation is an example of a dilemma faced by decision makers where design practices that can best serve the intended user base are disincentivized by the wider public health and medical research systems, resulting, in some respects, in a lower-quality HIT. The complexities and often contradicting incentives in the public health and medical research spheres can have the effect of frustrating progress in what should be the shared aim of improving public health outcomes. In such cases, marginalized populations that face multiple compounding barriers to access to quality care are the ones that are the most adversely affected, particularly in acute, abnormal situations such as natural disasters or a global pandemic [51]. To achieve the most impact, HIT solutions and software development resources should directly solve problems such as this that disproportionately impact marginalized populations. For example, one method of overcoming this problem could be to automate content moderation via natural language processing [52].

### Designing for Culturally Diverse Groups

The findings of this study suggest that although culturally diverse groups have specific concerns about design (eg, affirmation of diversity in sexual preference and gender identity and the provision of multiple language options), their needs fall broadly within universal design principles. For example, our LGBTQIA+ participants wanted a way to display their sexuality and pronouns alongside their dashboard of results. In addition to being able to represent themselves more fully, this would also increase their comfort levels with their health professional as they would not have to directly disclose this information face-to-face. The psychosocial context behind our participants’ recommendations is crucial and cannot be divorced from the recommendations themselves without running the risk of developing solutions that could alienate the culturally diverse groups from which they hail. For this reason, we have synthesized our findings into general rather than specific recommendations.

Textbox 1 provides general recommendations on designing HITs for mental health that were drawn from co-design with culturally diverse populations and based on self-determination theory. Specifically, designers should consider the intended functionality of the HIT and how the HIT can fulfill its users’ basic needs of autonomy, competence, and relatedness (by providing perceptions of control, usability, and affirmation). This would require identifying barriers to their access to, and adoption of, the technology (ideally through a co-design process similar to what was adopted in this study) and designing the technology to mitigate these barriers.

**General recommendations on designing health information technologies for mental health based on self-determination theory.**

1. Identify the purpose of the health information technology (HIT) and the mechanisms through which it achieves it aims
2. Consider how the HIT can fulfill users’ basic needs of autonomy, competence, and relatedness through providing perceptions of control, usability, and affirmation:
  Control includes the ability to control user experience as well as the ability to control user data
  Usability includes ease of use and understanding, the provision of meaningful guidance, and ease of integration into daily life (eg, interoperability)
  Affirmation includes users feeling both valued and included within the target user base
3. Consider the possible barriers to adopting the HIT, including access and use, from all potential user groups
4. Consider how to design the HIT to directly address as many adoption barriers as possible while still preserving perceptions of control, usability, and affirmation

Adoption barriers that this study and previous research have identified include young people’s lack of easy access to personal computers [53] as well as poor internet connectivity in many nonurban parts of Australia [54]. HIT designers should be aware of inequalities and how they compound and not perpetuate them. For example, they should neither enable discrimination nor systematically exclude groups of users who may not have adequate internet connectivity or reading level. Compassionate design that puts these barriers at the forefront of consideration is required to ensure that technologies can be accessed by all. Our study results underscore the importance of user testing with the target population to confirm that the technology satisfies their needs and to identify potential barriers that may prevent them from accessing these technologies.

### Limitations

A limitation of this study was that the researchers did not exclusively advertise to and recruit participants from the populations of interest within this study (Aboriginal and Torres Strait Islander people, LGBTQIA+ people, and CALD people in nonurban areas). Instead, all young people attending *headspace* (aged between 12 and 25 years), *headspace* staff, and supportive others were invited to participate in the workshops. Therefore, although participants who identified as being of these populations were present at workshops, the data collected cannot be exclusively attributed to these populations. Future research should consider recruiting participants exclusively from these populations and provide appropriate support to ensure comfort in communication (eg, an Aboriginal or Torres Strait Islander person to facilitate the workshop tailored to that population). Artifacts from the *headspace* Dubbo workshop (annotated screenshots and drawings of technology prototypes) were not included in the analysis for this study, but all researcher-scribed notes were included.

Our inclusion criteria would also have excluded CALD participants who were not proficient in English. Hence, our study results cannot be fully generalized to CALD communities. As mentioned above, future research should recruit exclusively from CALD populations and provide appropriate support (eg, by conducting workshops in participants’ native language or providing a translator).

Furthermore, our workshops included a mixed group of participants (young people, their supportive others, and health professionals). This gave facilitators the opportunity to address tensions and potential contradictions in what each of these groups wanted from an HIT for mental health. However, although workshop facilitators did their best to encourage open discussion, the implicit power difference among these groups could have discouraged this from participants belonging to less socially powerful groups (eg, young people). Similarly, workshops were not audiorecorded or videorecorded to facilitate discussion. However, this limited the details on which scribe notes could be taken.

Finally, these insights arise from a relatively narrow (though important) source, namely, representative end users, including young people, health professionals, and supportive others (our participants), further interpreted through the lens of independent knowledge translators (2 young Australian women) and mental health researchers (the authors). Although these groups have their own spheres of expertise, they are not the only important perspectives in the design of HITs for mental health. There are other considerations to account for in the design of such technologies, particularly in terms of specialist detail in user experience design, security, governance, and software engineering. As a result, the findings of this study should be taken as informed recommendations for how similar HITs can be structured as well as a validation of existing ideas only.

### Conclusions

Recent research has underscored the importance of iteratively testing the acceptability of HITs with the target end user population [25,33]. Through a series of co-design workshops, this study sought to extend the scope of our previous research from urban and nonurban Australian adolescents to include culturally diverse populations in nonurban areas of Australia. Although we identified several barriers and preferences specific to these populations in our co-design workshops, our results support the application of theory-based design of HITs (eg, self-determination theory) to develop user experiences that fulfill the universal basic psychological needs of autonomy, competence, and relatedness, through providing perceptions of control, usability, and affirmation. Deeper reflection on our findings also reveals the inherent tensions and difficulties in balancing the multiple, sometimes contradicting requirements of mental health technology stakeholders, including, but not limited to, health professionals, regulatory bodies, individual users, service managers, and best practice of the software development industry.

## Appendix

Multimedia Appendix 1 Example workshop agenda.

Multimedia Appendix 2 Full coding template and codebook of definitions.

## Abbreviations

BMC: Brain and Mind Centre
CALD: culturally and linguistically diverse
HIT: Health Information Technology
LGBTQIA+ people: People of diverse genders and sexualities, inclusive of and not limited to lesbian, gay, bisexual, transgender, queer, intersex, asexual, questioning, and pansexual people
YMH and Tech Program: Youth Mental Health and Technology Program

## References

[1] Bishop L, Random A, Laverty M, Gale L (2017). Mental health in remote and rural communities. Royal Flying Doctor Service of Australia.

[2] Lawrence David, Johnson Sarah E, Hafekost Jennifer, Boterhoven de Haan Katrina, Sawyer Michael G, Ainley John, Zubrick Stephen R (2016). The Mental Health of Children and Adolescents. Report on the second Australian Child and Adolescent Survey of Mental Health and Wellbeing. Department of Health.

[3] Brenes Gretchen A, Danhauer Suzanne C, Lyles Mary F, Hogan Patricia E, Miller Michael E (2015). Barriers to Mental Health Treatment in Rural Older Adults. Am J Geriatr Psychiatry.

[4] Fennell K, Hull M, Jones M, Dollman J (2018). A comparison of barriers to accessing services for mental and physical health conditions in a sample of rural Australian adults. Rural Remote Health.

[5] Seidler ZE, Rice SM, Dhillon HM, Cotton SM, Telford NR, McEachran J, Rickwood DJ (2020). Patterns of Youth Mental Health Service Use and Discontinuation: Population Data From Australia's Headspace Model of Care. Psychiatr Serv.

[6] NMHC (2014). The National Review of Mental Health Programmes and Services. National Mental Health Commission.

[7] Brown A, Rice SM, Rickwood DJ, Parker AG (2015). Systematic review of barriers and facilitators to accessing and engaging with mental health care among at-risk young people. Asia-Pacific Psychiatry.

[8] Hilferty F, Cassells R, Muir K, Duncan A, Christensen D, Mitrou F (2015). Is headspace making a difference to young people's lives? Final Report of the independent evaluation of the headspace program. Social Policy Research Centre, UNSW Australia.

[9] Rosenstreich G (2013). LGBTI People: Mental Health and Suicide. Revised 2nd Edition. National LGBTI Health Alliance.

[10] Hunter E (2007). Disadvantage and discontent: A review of issues relevant to the mental health of rural and remote Indigenous Australians. Aust J Rural Health.

[11] Zubrick S, Silburn S, Lawrence D, Mitrou F, Dalby R, Blair E (2005). The Western Australian Aboriginal Child Health Survey: The Social and Emotional Wellbeing of Aboriginal Children and Young People. The Western Australian Aboriginal Child Health Survey.

[12] Wohler Y, Dantas JA (2017). Barriers Accessing Mental Health Services Among Culturally and Linguistically Diverse (CALD) Immigrant Women in Australia: Policy Implications. J Immigr Minor Health.

[13] Cross W, Singh C (2014). Dual vulnerabilities: Mental illness in a culturally and linguistically diverse society. Contemporary Nurse.

[14] Kuipers P, Lindeman MA, Grant L, Dingwall K (2016). Front-line worker perspectives on Indigenous youth suicide in Central Australia: initial treatment and response. Advances in Mental Health.

[15] Hinton R, Kavanagh DJ, Barclay L, Chenhall R, Nagel T (2015). Developing a best practice pathway to support improvements in Indigenous Australians’ mental health and well-being: a qualitative study: Figure 1. BMJ Open.

[16] Kilian A, Williamson A (2018). What is known about pathways to mental health care for Australian Aboriginal young people?: a narrative review. Int J Equity Health.

[17] Smith E, Jones T, Ward R, Dixon J, Mitchell A, Hillier L (2014). From Blues to Rainbows: Mental health and wellbeing of gender diverse and transgender young people in Australia. he Australian Research Centre in Sex, Health, and Society.

[18] Robinson K, Bansel P, Denson N, Ovenden G, Davies C (2014). Growing Up Queer: Issues Facing Young Australians Who Are Gender Variant and Sexuality Diverse. Young and Well Cooperative Research Centre.

[19] Minas H, Kakuma R, Too L, Vayani H, Orapeleng S, Prasad-Ildes R, Turner G, Procter N, Oehm D (2013). Mental health research and evaluation in multicultural Australia: developing a culture of inclusion. Int J Ment Health Syst.

[20] de Anstiss H, Ziaian T, Procter N, Warland J, Baghurst P (2009). Help-seeking for Mental Health Problems in Young Refugees: A Review of the Literature with Implications for Policy, Practice, and Research. Transcult Psychiatry.

[21] Forbes-Mewett H, Sawyer A (2011). Mental health issues amongst international students in Australia: Perspectives from professionals at the coal-face. Threadgold S, Kirby E. editors.

[22] Thompson T, Brailer D (2004). The Decade of Health Information Technology: Delivering Consumer-centric and Information-rich Health Care. Framework for Strategic Action.

[23] Price M, Yuen EK, Goetter EM, Herbert JD, Forman EM, Acierno R, Ruggiero KJ (2014). mHealth: a mechanism to deliver more accessible, more effective mental health care. Clin Psychol Psychother.

[24] Hickie IB, Davenport TA, Burns JM, Milton AC, Ospina-Pinillos L, Whittle L, Ricci CS, McLoughlin LT, Mendoza J, Cross SP, Piper SE, Iorfino F, LaMonica HM (2019). Project Synergy: co-designing technology-enabled solutions for Australian mental health services reform. Med J Aust.

[25] Fleming TM, de BD, Khazaal Y, Gaggioli A, Riva G, Botella C, Baños RM, Aschieri F, Bavin LM, Kleiboer A, Merry S, Lau HM, Riper H (2016). Maximizing the Impact of e-Therapy and Serious Gaming: Time for a Paradigm Shift. Front Psychiatry.

[26] Pretorius C, Chambers D, Cowan B, Coyle D (2019). Young People Seeking Help Online for Mental Health: Cross-Sectional Survey Study. JMIR Ment Health.

[27] Rowe SC, Davenport TA, Easton MA, Jackson TA, Melsness J, Ottavio A, Sinclair J, Hickie AM IB (2020). Co‐designing the InnoWell Platform to deliver the right mental health care first time to regional youth. Aust. J. Rural Health.

[28] Hickie IB, Davenport TA, Burns JM, Milton AC, Ospina-Pinillos L, Whittle L, Ricci CS, McLoughlin LT, Mendoza J, Cross SP, Piper SE, Iorfino F, LaMonica HM (2019). Project Synergy: co-designing technology-enabled solutions for Australian mental health services reform. Med J Aust.

[29] Ospina-Pinillos L, Davenport TA, Ricci CS, Milton AC, Scott EM, Hickie IB (2018). Developing a Mental Health eClinic to Improve Access to and Quality of Mental Health Care for Young People: Using Participatory Design as Research Methodologies. J Med Internet Res.

[30] Hagen P, Collin P, Metcalf A, Nicholas M, Rahilly K, Swainston N (2012). Participatory Design of evidence-based online youth mental health promotion, prevention, early intervention and treatment.

[31] Yarosh S, Schueller SM (2017). “Happiness Inventors”: Informing Positive Computing Technologies Through Participatory Design With Children. J Med Internet Res.

[32] Jessen S, Mirkovic J, Ruland CM (2018). Creating Gameful Design in mHealth: A Participatory Co-Design Approach. JMIR Mhealth Uhealth.

[33] Sockolow P, Schug S, Zhu J, Smith T, Senathirajah Y, Bloom S (2016). At-risk adolescents as experts in a new requirements elicitation procedure for the development of a smart phone psychoeducational trauma-informed care application. Informatics for Health and Social Care.

[34] Ospina-Pinillos L, Davenport T, Mendoza Diaz A, Navarro-Mancilla A, Scott EM, Hickie IB (2019). Using Participatory Design Methodologies to Co-Design and Culturally Adapt the Spanish Version of the Mental Health eClinic: Qualitative Study. J Med Internet Res.

[35] Ospina-Pinillos L, Davenport T, Navarro-Mancilla A, Cheng V, Cardozo AA, Rangel A Involving end users in adapting the Spanish version of the Mental Health eClinic for young people in Colombia: A pilot study using participatory design methodologies. JMIR.

[36] Cheng VWS, Davenport TA, Johnson D, Vella K, Mitchell J, Hickie IB (2018). An App That Incorporates Gamification, Mini-Games, and Social Connection to Improve Men's Mental Health and Well-Being (MindMax): Participatory Design Process. JMIR Ment Health.

[37] Bauer M, Gaskell G (2000). Qualitative Researching with Text, Image and Sound. London: SAGE Publications Ltd.

[38] Braun V, Clarke V (2019). Reflecting on reflexive thematic analysis. Qualitative Research in Sport, Exercise and Health.

[39] King N, Brooks J, Tabari S (2018). Template Analysis in Business Management Research. Qualitative Methodologies in Organization Studies: Volume II: Methods and Possibilities.

[40] Moltu C, Stefansen J, Svisdahl M, Veseth M (2012). Negotiating the coresearcher mandate - service users' experiences of doing collaborative research on mental health. Disabil Rehabil.

[41] Deci EL, Ryan RM (2000). The "What" and "Why" of Goal Pursuits: Human Needs and the Self-Determination of Behavior. Psychological Inquiry.

[42] Ryan R, Deci E (2002). Overview of self-determination theory: An organismic dialectical perspective. Handbook of self-determination research.

[43] Przybylski AK, Rigby CS, Ryan RM (2010). A motivational model of video game engagement. Review of General Psychology.

[44] Ryan RM, Rigby CS, Przybylski A (2006). The Motivational Pull of Video Games: A Self-Determination Theory Approach. Motiv Emot.

[45] Pretorius C, Chambers D, Coyle D (2019). Young People's Online Help-Seeking and Mental Health Difficulties: Systematic Narrative Review. J Med Internet Res.

[46] Pretorius C, McCashin D, Kavanagh N, Coyle D (2020). Searching for Mental Health: A Mixed-Methods Study of Young People's Online Help-seeking. CHI '20.

[47] Cheek C, Fleming T, Lucassen MF, Bridgman H, Stasiak K, Shepherd M, Orpin P (2015). Integrating Health Behavior Theory and Design Elements in Serious Games. JMIR Mental Health.

[48] Lister C, West JH, Cannon B, Sax T, Brodegard D (2014). Just a fad? Gamification in health and fitness apps. JMIR Serious Games.

[49] Mayer G, Gronewold N, Alvarez S, Bruns B, Hilbel T, Schultz J (2019). Acceptance and Expectations of Medical Experts, Students, and Patients Toward Electronic Mental Health Apps: Cross-Sectional Quantitative and Qualitative Survey Study. JMIR Ment Health.

[50] Deterding S (2015). The Lens of Intrinsic Skill Atoms: A Method for Gameful Design. Human–Computer Interaction.

[51] Bowleg L (2020). We’re Not All in This Together: On COVID-19, Intersectionality, and Structural Inequality. Am J Public Health.

[52] Hussain MS, Li J, Ellis LA, Ospina-Pinillos L, Davenport TA, Calvo RA, Hickie IB (2015). Moderator Assistant: A Natural Language Generation-Based Intervention to Support Mental Health via Social Media. Journal of Technology in Human Services.

[53] Ellis LA, Collin P, Davenport TA, Hurley PJ, Burns JM, Hickie IB (2012). Young men, mental health, and technology: implications for service design and delivery in the digital age. J Med Internet Res.

[54] Orlowski S, Lawn S, Antezana G, Venning A, Winsall M, Bidargaddi N, Matthews B (2016). A Rural Youth Consumer Perspective of Technology to Enhance Face-to-Face Mental Health Services. J Child Fam Stud.

