# Supplementary material for: Recommendations for Designing Health Information Technologies for Mental Health Drawn From Self-Determination Theory and Co-design With Culturally Diverse Populations: Template Analysis
Source: J Med Internet Res. 2021 Feb 10;23(2):e23502. doi: 10.2196/23502 (PMC7904400; doi:10.2196/23502)
Supplement: Multimedia Appendix 1 [file jmir_v23i2e23502_app1.docx]

**Participatory Design Workshop Agenda - Example**

**Focus** FGG Evaluation of BMC Youth Platform Technology, and User Journeys

**Date** *[enter date]*

**Time** *[enter time]*

**Location** *[enter location]*

**BMC Facilitator** *[names]*

**BMC Scribe** *[name]*

**Counselling Support** *[names]*

**Materials required**

- Participant Information Statements
- Participant Consent Forms
- Screener survey
- Pens or pencils
- Post notes
- Sketch pad
- Name tags
- Wireframes of BMC Youth Platform
- Butchers paper

**AGENDA ITEMS**

- Consent

**4:00pm Introduction**

- Welcome, Acknowledgement to Country
- Introductions
- Repeat group consent
- Overview of workshop - *what is participatory design?*
- Icebreaker exercise

**4:10pm Discovery (10 mins)**

Prompted discussion **(casual)** –

- *What is your favourite piece of technology? (e.g. PS4 or Facebook?)*
- *Do you ever have trouble with internet connection? What do you do if you cannot connect to the internet?*

**4:20pm Evaluation (15 mins)**

Introduction of BMC Youth Platform

- Participants are introduced to BMC Youth Platform, via an A3 hand out which includes an overview of the technology:

**1. Get started**

complete an online assessment

**2. Understand your needs**

see your results on a dashboard

**3. Get the right recommendations for you**

support for your mental health and wellbeing

**4. See how you’re improving**

track your progress over time

**5. Collaborate with your clinician**

working together on your health care plan

- Participants are given an opportunity to ask questions about the BMC Youth Platform to ensure an understanding of the concept
- Participants are given 2-3mins with each new screen to write their initial look, feel, understanding of the content on the page.

**4.35pm Discovery (introduction to prototyping) (15mins)**

Technology use

*The co-design nature of BMC Youth Platform is explained to participants. i.e. In order to further develop this technology, ‘’now we need your help”. “We’re interested to hear about your experiences with using technology to support your mental health”.*

- Are you aware of your health and wellbeing?
- Do you notice changes in your health and wellbeing?
- Do you track your health and wellbeing? If you do, what do you use?
- Have you ever used technology to support your health/mental health? What do you use? How do you use it? Do you have access to it?
- If you don’t use technology, why not? What do you use to track your health if you don’t use technology?
- Do you/would you use technology in your care at headspace? (E.g. use an app or etool with your clinician to track progress) (Clinicians: do you use technology with your clients?)
- If you or someone you know needs mental health support, what services would you engage with to get the help you need? Do you prefer using one technology over another? (phone, online chat, etc)
- What is your experience using a dashboard? What do you like? Want to change?
- Would you use the dashboard to share information? Who with? How?

**4:50pm Prototype**

Exploring the dashboard:

- Participants are introduced to more detail about the BMC Youth Platform, and are given an A3 hand out of three possible screens:

1) Dashboard of health cards 2) Health history 3) Care options

- Participants are again informed about the purposes of each of these components of the system. As a group, particpants provide feedback on, and brainstorm new ideas for these tabs. Ideas are written on butchers paper attached to the wall (or a whiteboard).

Possible questions:

- *What would you expect to see on this page?*
- *What should/should not be included?*
- *How would you like to see the information displayed?*
- *What do you think of health card names? (dashboard specific)*
- *How would you use this page?*
- *Are you comfortable entering personal information and displaying this information on this page? What might you not feel comfortable to display?*

**6:55pm Question time/wrap up (5 mins)**
