# Supplementary material for: Recommendations for Designing Health Information Technologies for Mental Health Drawn From Self-Determination Theory and Co-design With Culturally Diverse Populations: Template Analysis
Source: J Med Internet Res. 2021 Feb 10;23(2):e23502. doi: 10.2196/23502 (PMC7904400; doi:10.2196/23502)
Supplement: Multimedia Appendix 2 [file jmir_v23i2e23502_app2.docx]

# Full Coding Template and ‘Codebook’ of Definitions.

| **Theme** | **Notes** |
| --- | --- |
| Ability to control own user experience | Importance of being able to control your experience of using the technology   - How the technology looks: active customisation of colours, layout, language, avatar, font size, etc. - How the technology works: care options, organising how your data is presented, etc. - Ability to provide user feedback that will be actioned on |
| Ability to control own data | Importance of being able to control how the technology treats your data and your health   - Data security - Data privacy and sharing (control who sees what information) - Able to provide as much or as little information as is comfortable |
| Technology should be easy to use and understand | Technology should be easy to use and understand at first glance – not overwhelming or frustrating to use   - Usability including layout, icons, and imagery - Ensure language is clear, unambiguous, and consistent   - Avoid difficult clinical jargon |
| Technology should provide meaningful guidance | Technology should provide meaningful guidance on how to use and interpret the Platform   - Instructional prompts (including reminder prompts) on what to expect and how that will help user - More information on features and clinical domains that user can access (psychoeducation) - Help button/FAQ - Provide information in meaningful chunks and not overwhelm so as to support understanding (appropriate presentation of information)   - e.g. not too much text, showing most important domains first on dashboard, etc. - Gradual onboarding |
| Technology should be easy to integrate into daily life | Technology should be easy to integrate into daily life   - Integration with other apps, e.g. health apps or authenticating via social media - Mobile integration as young people do not use email or computers as frequently |
| Importance of making users feel valued | Importance of feeling comfortable in the technology – not judged   - Technology design should celebrate non-clinical aspects of personhood as well such as likes and aspirations - Positive framing celebrating achievements and strengths - Technology should not reduce individual to a diagnosis - Element of fun: e.g. gamification   Importance of making user not feel alienated   - Social connection with peers (even if indirect) - Normalising suboptimal mental health status (“It’s OK to feel bad”) - Using casual language and avoiding loaded terms |
| Importance of making users feel represented | Importance of feeling represented by the technology   - Technology design should make users feel comfortable and catered to regardless of demographic or clinical aspects such as sexuality, culture, or mental health status - Users should be able to provide more detailed information about themselves should technology limitations not represent them adequately e.g. new domains such as family violence, or subjective perception of own mental health, or elaborating on responses via free text   Importance of feeling catered to by the technology   - Technology should offer users a user experience (e.g. questionnaires, care options, native language support, etc.) that is **tailored** to their personal circumstances e.g. gender, culture, location - Technology should be usable regardless of physical or mental ability (mental health status, disability, or other accessibility factors) - Technology should acknowledge ways different cultures approach mental health (‘life circumstances’ vs ‘mental illness’) |
| Barriers to adoption | - Internet and data limitations - Financial cost and hardware limitations - Healthcare system e.g. limited amount of sessions - Service using other competing software |
| Technology supporting care coordination and delivery [concrete examples of how health services can use technology to enhance care] | Technology can help health professional and client clearly understand the progression of the client’s care   - Technology can profile health professionals to give users idea of what to expect before appointments - Technology can track client’s health over a variety of domains (routine outcome monitoring and holistic view of health) - Technology keeps a record of care options and health status over time - Technology can prompt topics for conversation during appointments relating to the client’s health status   Technology can support health professional and client in directing care   - Health professional can tailor their support for the client according to their health status - Client can make informed choices on which health domains and care options to focus on based on their own health status   Technology can improve efficiencies in clinical care   - Synchronous communications e.g. video calling or web chat - Clinical governance concerns - Risk management in emergency scenarios - Routine clinical procedures e.g. transfers - Easier to complete questionnaire on device than face-to-face |
